# piRNAs as Potential Regulators of Mammary Gland Development and Pathology in Livestock

**DOI:** 10.3390/vetsci12060594

**Published:** 2025-06-17

**Authors:** Wenjing Yu, Zixuan Zhang, Zhonghua Wang, Xusheng Dong, Qiuling Hou

**Affiliations:** College of Veterinary Medicine, Basic Veterinary Medicine, Panhe Campus, Shandong Agricultural University, Taian 271018, China; yuwenjing0224@163.com (W.Y.); zzx11012025@163.com (Z.Z.); zhwang@sdau.edu.cn (Z.W.)

**Keywords:** piRNAs, mammary development, mastitis, cow

## Abstract

PiRNAs are a subclass of non-coding RNAs, 26–31 nucleotides (nt) in length, that form regulatory complexes through their interaction with PIWI proteins. Studies in model organisms have demonstrated that piRNAs play crucial roles in tissue development and in predicting disease outcomes, positioning them as promising targets for developmental regulation and therapeutic intervention. In contrast, research on piRNAs in animal husbandry is still in its early stages and has not received sufficient attention. Despite this, the few studies available in livestock research have revealed that piRNAs serve as key regulators of reproductive development, underscoring their significant regulatory potential in farm animals and justifying further investigation. Accordingly, this review uses the bovine mammary gland as an exemplary case to summarize the progress in piRNA research related to mammary development and disease. The role of piRNAs in regulating breast cancer stem cell proliferation and modulating inflammatory progression is a highly active area of research. We hypothesize that piRNAs may play a potential role in regulating both mammary gland development and mastitis, making them promising targets for enhancing mammary development and overall health in dairy cattle and providing a theoretical foundation for further piRNA applications in animal husbandry.

## Simple Summary

PIWI-interacting RNAs (piRNAs) have been demonstrated to maintain transposon silencing and regulate reproductive development and disease progression by binding to PIWI proteins. However, research on piRNAs in livestock animals is still in its infancy, with only preliminary studies available regarding their roles in the reproduction of swine, bovine, and ovine species. Given the regulatory mechanisms of piRNAs in mammary cancer and inflammation, as well as the established roles of other non-coding RNAs (ncRNAs) in mammary development, this paper focuses on the dairy cow mammary system to explore the potential functions of piRNAs in mammary development and mastitis. This review offers a novel perspective on mammary gland biology in livestock and provides a theoretical foundation for understanding the role of piRNAs in mammary gland development and associated diseases, representing significant value for both basic research and practical applications.

## 1. Introduction

The livestock farming industry confronts a dual challenge of global population growth and an increasing demand for food. This dual challenge entails enhancing production efficiency and adopting green, circular development practices. Consequently, this imposes increased demands on fundamental aspects such as genetic enhancement, disease prevention and control, and reproductive efficiency. In this context, epigenetic regulatory mechanisms have emerged as a promising research field with the potential to alleviate production bottlenecks in livestock, owing to their crucial roles in gene expression regulation and transgenerational inheritance [1].

The impact of epigenetic regulatory networks on key phenotypic traits—including growth performance, stress tolerance, and reproductive efficiency—in livestock is well documented. This regulation is mediated through DNA methylation, histone modifications, and ncRNAs, whose significance in livestock is increasingly recognized [2,3]. Among these, DNA methylation has been identified as a prominent feature of heat stress in dairy cows. It has been demonstrated that the exposure of bovines to elevated ambient temperatures during the period of gestation results in the occurrence of differential DNA methylation in utero, as well as intrauterine growth restriction of the offspring, which is partially responsible for long-term phenotypic alterations [4]. It was demonstrated that heat stress resulted in a significant increase in the methylation level of the Dual Leucine Zipper Kinase-like (DNLZ) promoter in dairy cows. This finding implies that the changes could be used as an epigenetic marker for heat tolerance traits and provide direction for breeding heat-resistant dairy cows [5]. In mastitis regulation, the presence of differentially expressed miRNAs in the milk exosome from healthy cows and cows with mastitis suggests that such molecules may serve as a significant research tool for studying the molecular mechanisms of mastitis in dairy cows [6]. Furthermore, differentially expressed lncRNAs were identified in the mammary tissues of cows in the early and non-lactating stages of lactation. Through pathway analyses, the authors showed that these differentially expressed lncRNAs may be involved in important signaling cascades and regulatory processes associated with immune responses, cell growth, and intracellular signaling [7]. Despite the present focus on DNA methylation and histone modification in livestock epigenetic studies, the importance of ncRNAs is becoming increasingly recognized as research in this area progresses. Consequently, a comprehensive understanding of ncRNAs is of great value for the development of animal husbandry.

NcRNAs include a variety of forms, such as microRNAs (miRNAs), small interfering RNAs (siRNAs), piRNAs, and long non-coding RNAs (lncRNAs), among others. Of particular note is the discovery of piRNAs in 2006 [8,9,10,11,12], which have been shown to maintain genome stability and regulate protein synthesis by binding to PIWI proteins [13,14]. In addition to this, piRNAs have been shown to play a crucial role in the maintenance of normal gonadal development [15]. Recently, the significance of piRNAs in inflammatory responses and cancer research, particularly in breast cancer, has gained increasing recognition. This nc RNA is emerging as potential prognostic markers and therapeutic targets for breast cancer [16,17].

At the present time, research into the mechanism of piRNAs has been primarily focused on model organisms such as nematodes, Drosophila, and mice. To date, studies pertaining to animal husbandry have exclusively encompassed a proportion of the expression profiles, and no in-depth mechanistic studies have been undertaken to date. The present focus of research in the field of piRNAs is on cancer and inflammation, where the role of these molecules is to regulate the proliferation of breast cancer cells [18], as well as to inhibit inflammatory signaling pathways and thus regulate inflammatory progression [19]. The studies of piRNAs in the specific inflammatory response of mastitis or in mammary development have not been found. In this paper, we present a systematic review of the production, characteristics, and functions of piRNAs, as well as their role in inflammation and breast cancer, and propose a hypothesis regarding their potential roles in mammary gland development and mastitis with the generalization of the inflammatory response and the characteristics of mammary gland development. Additionally, we provided a concise overview of the prospects for the utilization of piRNAs in domestic animals. To explore the potential roles of piRNAs in mammary development and disease through a review of their functions and mechanisms, providing new directions for research in livestock biology.

## 2. The Process of Generating piRNAs

As a class of ncRNAs, piRNAs exhibit a unique biogenesis pathway that encompasses both primary processing and secondary amplification (also known as the ping-pong cycle, a self-reinforcing amplification loop of piRNA production). The process of piRNA biogenesis is found to be identical in the majority of animal germ cells [20]. Notably, piRNA biogenesis occurs independently of the Dicer enzyme. Primary piRNAs are characterized by a single-stranded linear structure featuring a 5′-monophosphate and a 2′-O-methyl modification at the 3′ end [21]. In contrast, secondary piRNAs display a 10 nt complementarity at their 5′ ends with primary piRNAs [22]. This characteristic underpins the efficient repression of transposons by the piRNA pathway.

### 2.1. Primary Processing

Primary processing represents a crucial stage in piRNA biogenesis. The sources of piRNAs include transposon-derived piRNAs, lncRNA-derived piRNAs, and mRNA-derived piRNAs [22]. The primary processing of piRNAs is a complex process that involves multiple proteins, whose identities vary across species (Table 1). In Drosophila, the Rhino−Deadlock−Cutoff (RDC) complex, located in the nucleus, plays a pivotal role in recognizing heterochromatin regions. Subsequently, the RDC complex recruits the Moonshiner protein, activates RNA polymerase II (Pol II), and initiates the transcription of piRNA clusters from both genomic strands. These processes culminate in the production of either sense or antisense piRNA precursors (pre-piRNA) [23]. The pre-piRNA is subsequently cleaved by the 5′-terminal enzyme—Zucchini (Zuc) protein, typically at a uracil-enriched region, resulting in piRNAs with a strong 5′ uracil bias (1U bias) [24]. The cleaved precursor RNAs are trimmed by the 3′-terminal enzyme—Trimmer protein to yield single-stranded, linear piRNAs with a final length of 26–31 nt, thereby forming primary piRNAs [25,26,27,28].

### 2.2. Secondary Amplification

**Figure 1:**
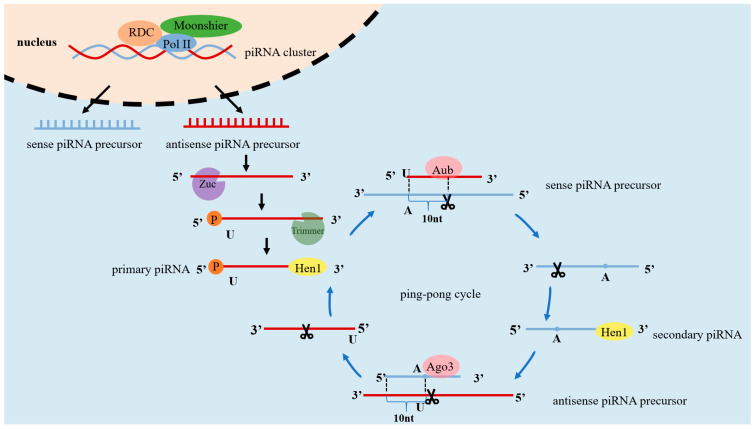
The primary and ping-pong mechanism for piRNA biogenesis.

Secondary amplification, also known as the “ping-pong cycle,” represents a crucial stage in piRNA biogenesis, resulting in an increased production of piRNAs through synergistic interactions with diverse PIWI proteins [32,37]. In Drosophila, for instance, primary antisense piRNAs bind to the Aub protein to form the piRNA-Aub complex, which exhibits endonuclease activity. This complex subsequently targets the sense pre-piRNA through a process of base complementary pairing, then trimmed by exonucleases and modified by hua enhancer 1 (HEN1) methyltransferase, resulting in the formation of the secondary piRNA. The 5′ end of the secondary piRNA begins at the 10th nt from the cleavage site of the target RNA, which typically features an adenine residue (10A bias) [37,38,39]. The secondary piRNA then associates with the Ago3 protein, forming a complex that targets antisense pre-piRNA through base-pairing. The Ago3 complex cleaves the target RNA, thereby generating a new antisense pre-piRNA [40]. The 5′ end of this new antisense pre-piRNA begins at the 10th nt from the cleavage site—typically featuring a uracil residue—and, after exonuclease trimming and modification by HEN1 methyltransferase, matures into a new primary antisense piRNA [41]. The newly formed primary antisense piRNA then re-associates with the Aub protein, initiating another round of the ping-pong cycle; this cycle is represented schematically in Figure 1.

## 3. Characteristics and Functions of piRNAs

The principal function of piRNAs is believed to involve their binding to PIWI proteins, thereby forming functional piRNA–PIWI complexes. Their main functions include silencing transposons to ensure genomic stability, preserving fertility, and regulating disease development. In early studies, research focused on the mechanism of piRNA silencing of transposable factors at the transcriptional and post-transcriptional levels in animal germ cells. This is closely related to the regulatory function of reproductive development [22,42]. Subsequent evidence has shown that piRNAs can also regulate reproductive development by regulating the protein-coding genes involved in embryogenesis [43,44]. More recent studies have focused on the regulatory roles of piRNAs in inflammation and breast cancer, with their expression being modulated by environmental, nutritional, stress-related, pathological, and genetic factors, as well as by maternal transgenerational effects [45,46].

### 3.1. The Characteristics of piRNAs

Within the non-coding RNA families, piRNAs, miRNAs, and siRNAs exhibit significant disparities in their generation mechanisms, action proteins, and functional levels, despite their common classification as small molecule RNAs.

In contrast to the biogenesis of miRNAs and siRNAs, which is Dicer-dependent, piRNA generation occurs independently of Dicer. It is initiated from a dedicated piRNA cluster and non-cluster templates, resulting in the production of mature piRNAs (typically 26–31 nt in length) through primary processing and secondary amplification [22]. In contrast, miRNA biogenesis initiates with the transcription of primary miRNA transcripts (pri-miRNAs) by Pol II. These pri-miRNAs fold into characteristic hairpin structures within the nucleus, where they are recognized and cleaved by Drosha. This processing generates precursor miRNAs (pre-miRNAs), which are subsequently processed by the cytoplasmic RNase III enzyme Dicer into miRNA duplexes with a length of 21–24 nt. One strand of the duplex is selectively loaded onto AGO subfamily proteins to form the functional RNA-induced silencing complex (RISC) [47]. Similar to miRNAs, siRNAs originate from exogenous viral genomes or endogenous repetitive sequences, which are transcribed into long double-stranded RNAs (dsRNAs). These dsRNAs undergo precise cleavage by the ribonuclease Dicer, generating short RNA duplexes, which are 20–24 nt long. Following processing, one strand of the duplex (the guide strand) is selectively incorporated into AGO subfamily proteins within the RISC [48,49]. Secondly, it is important to note that the binding proteins of piRNAs are distinct from those of miRNAs and siRNAs. The silencing of small molecule RNAs has been observed to interact with Argonaute family proteins, which constitute the core part of RNA-induced silencing [50]. Argonaute proteins have been identified as multidomain proteins, primarily comprising the PAZ and PIWI domains, and encompassing both AGO subfamily and PIWI subfamily proteins [51,52]. It has been demonstrated that, in contrast to the interaction of miRNAs and siRNAs with AGO subfamily proteins, piRNAs interact with the PIWI subfamily proteins [53].

**Figure 2:**
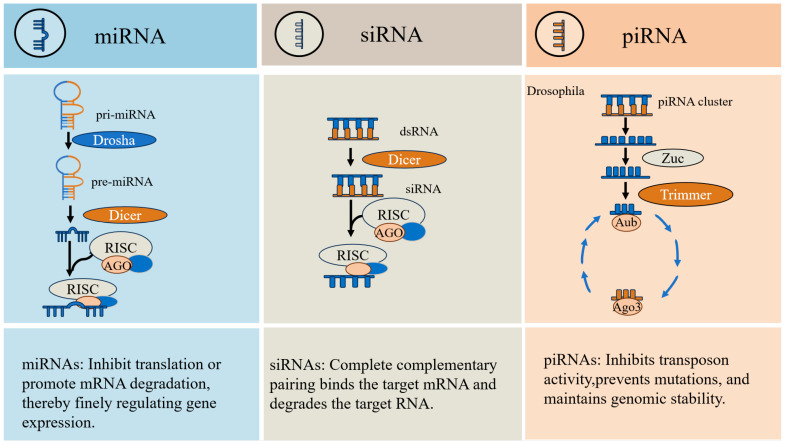
Differences between miRNA, siRNA, and piRNA.

In conclusion, the core functions of piRNAs are distinct from those of miRNAs and siRNAs. The primary function of piRNAs is to maintain the stability of the germ cell genome, primarily through the process of transposon silencing. This process involves the modification of transposon genes at the transcriptional level, resulting in the suppression of their expression (e.g., DNA methylation and histone modification). Furthermore, at the post-transcriptional level, the process directly cleaves transposon-originated RNA molecules in order to regulate gene expression [54,55,56]. Meanwhile, miRNAs play a pivotal role in regulating gene expression at the post-transcriptional level. By binding to the 3′ untranslated region (3′UTR) of target mRNAs, miRNAs either inhibit translation via partial complementary pairing or promote mRNA deadenylation and subsequent degradation [57]. The siRNA is fully complementary to the target mRNA and functions by mediating RISC cleavage of the mRNA, resulting in degradation and subsequent gene silencing [58]. A comparative analysis reveals significant disparities among these classes (Figure 2). Notably, the investigation of piRNAs in livestock is a relatively recent development compared to research on other ncRNAs.

### 3.2. The Role of piRNAs in Silencing Transposons and Stabilizing Genomes

The piRNA pathway is critical for maintaining transposon silencing and sustaining gene stability, and is centrally dependent on the integrity of PIWI proteins. It has been demonstrated in model animals that piRNAs interact with PIWI proteins to generate recognition complexes which silence distinct transposons Tc3, GypsyDR1, long interspersed nuclear element-1 (L1), etc. [30,54]. This process is dependent on the RNA-dependent RNA polymerase (RdRPase) for the synthesis of 22-nucleotide guanine-starting RNA (22G-RNA), which in turn maintains long-term silencing through the worm-specific Argonaute (WAGO) pathway [59]. The absence of PIWI proteins (e.g., PRG-1, ZIWI, or PIWI) has been demonstrated to result in transposon activation, reduced germ cell numbers, and gonadal hypoplasia [31,38,60]. It has been established that certain proteins—defective P granules and sterile (DEPS-1), four ankyrin repeats, a sterile alpha motif, and leucine zipper 1 protein (Asz1), Maelstrom (Mael), etc.—are indispensable for the maintenance of complex structure or function, and their absence directly disrupts transposon repression [15,61,62].

The repression of transposons by piRNAs can be achieved through epigenetic mechanisms. It has been established that MILI and MIWI2, in conjunction with DNA methyltransferase 3-like (DNMT3L), are indispensable for the initiation of methylation processes. Furthermore, it has been determined that L1 and intracisternal A particle (IAP) play a pivotal role in this process. In the absence of MILI/MIWI2, there is a concomitant reduction in the level of transposon CpG methylation, resulting in the activation of the transposons [63,64]. 22G-RNA, in conjunction with the WAGO family protein heritable RNA interference (RNAi) defective-1 (HRDE-1) and the RNAi pathway protein nuclear RNAi defective-2 (NRDE-2), has been shown to collaborate in the recruitment of histone methyltransferase SET-25/32, thereby facilitating the catalysis of histone H3 lysine 9 trimethylation (H3K9me3) modification and, consequently, resulting in transcriptional repression through the establishment of heterochromatin structures by the heterochromatin protein like-2 (HPL-2). Concurrently, 22G-RNA has been demonstrated to directly mediate the degradation of target mRNAs and enhance the silencing effect [65]. Abnormal PIWI function has been demonstrated to reduce the level of H3K9me modification and heterochromatin protein 1 (HP1) enrichment, whilst concomitantly increasing the level of histone H3 lysine 4 methylation (H3K4me2/3) modification. This has been shown to inhibit chromatin formation in the transposons and its surrounding regions, thereby ultimately activating the transposons [66,67,68].

In addition to epigenetic regulation, it has been established that piRNAs are capable of obstructing the process of translation of transposon mRNAs through their capacity to bind to the 3′UTR region of the transposon mRNAs. Furthermore, it has been demonstrated that chromatin assembly factor 1 (CAF1) interacts with the MIWI/piRNA complex, thereby inducing the decay of the target mRNA [44], which further enhances the silencing effects on transposons. A significant number of studies have been conducted on model animals such as nematodes, Drosophila, and mice. However, there is a paucity of mechanistic studies of piRNAs in livestock animals.

### 3.3. Physiological Functions of piRNAs

The piRNA pathway is paramount for reproductive development, playing a critical role in safeguarding gametogenesis and maintaining fertility. At the level of chromosome dynamics, piRNAs play a pivotal role in assembling telomere protection complexes, thereby ensuring genomic stability [69]. Furthermore, the piRNA pathway directly influences chromosome condensation and segregation. In Drosophila germ cells, mitotic bodies composed of piRNA pathway proteins have been observed to bind to pericentromeric, piRNA-producing loci, thereby regulating condensing loading. Mutations in the piRNA pathway lead to aberrant condensing loading, causing delays in chromosome condensation and segregation defects [70]. The role of PIWI proteins in maintaining germline stem cells (GSCs) is underscored by observations showing that the deletion of PIWI results in reduced GSC numbers, whereas its overexpression enhances stem cell division [71]. Notably, mice deficient in the piRNA pathway protein Miwi2 exhibit a significant reduction in germ cell numbers with age [64]. The importance of fertility maintenance is further evidenced by studies in mice showing that mitochondrial phospholipase D (MitoPLD) gene dysfunction disrupts the piRNA biogenesis pathway, resulting in spermatogonial meiotic arrest [72]. In golden hamsters, piRNAs are essential for the development of spermatogonia and the formation of fertile oocytes [73]. Thus, the aberrant expression of genes associated with the piRNA pathway leads to the unscheduled activation of transposons in germ cells, meiotic arrest, impaired spermatogenesis and oogenesis, disrupted early embryonic development, and ultimately infertility. The above findings suggest that the piRNA pathway plays a pivotal role in preserving developmental potential and reproductive health.

### 3.4. Factors Regulating piRNAs

The expression of piRNAs is regulated by a multitude of factors. Recent studies have demonstrated in model animals that elevated temperatures convert Drosophila piRNA clusters from an inactive to an active state, leading to the generation of novel piRNAs. These novel piRNAs are characterized by enhanced stability and can be maternally inherited by subsequent generations [74]. Conversely, elevated temperatures have also been shown to decrease piRNA levels and reduce offspring fitness in C. elegans. However, following bacterial infection, the restoration of piRNA levels correlates with improved offspring fitness, suggesting that the piRNA pathway can dynamically respond to environmental signals and exert a lasting influence on progeny [75]. Altered nutritional levels modulate piRNA expression; for instance, differential piRNA profiles have been observed in the sperm of obese versus lean males [76]. Similarly, a Western-style diet has been found to alter piRNA expression in the testes of male mice [77], while a high-fat diet influences sperm piRNA profiles in both males and their offspring, subsequently impacting offspring metabolic function [78]. Furthermore, the present study demonstrates that short-term endurance training induces reversible changes in sperm piRNA expression [79]. Early traumatic stress has been shown to significantly downregulate piRNA cluster 110 in male mouse sperm [80]. Furthermore, microcystin–leucine–arginine (MC-LR) exposure has been reported to alter piRNA expression in the testes and prostate of offspring, leading to decreased testicular indices and prostate hyperplasia in male mice [45,81].

Consequently, fluctuations in environmental factors, stress levels, and nutritional intake can result in alterations in piRNA expression, which may be transmitted to subsequent generations. In the context of livestock production, environmental stressors—including nutritional imbalances and heat stress—often have detrimental effects on animal health and performance. Given the role of piRNAs in responding to environmental and nutritional cues, it is hypothesized that they may hold considerable potential for enhancing livestock productivity and resilience. We propose the following pivotal research questions: Can piRNAs be used as biomarkers of heat stress resilience in dairy cows? Can piRNA expression levels influence mammary gland development?

## 4. The Role of ncRNAs in Mammary Gland Development

The function of piRNAs as a class of ncRNAs in the development of the mammary gland remains to be elucidated; however, the role of other ncRNAs in this process has been the subject of recent research. In dairy farming, a cow’s economic value depends on its milk yield, which is driven by mammary gland development and lactation physiology. The mammary gland, a distinctive mammalian organ, displays diverse morphologies across different developmental stages—from embryonic morphogenesis and pubertal ductal expansion to gestational alveolar differentiation. Within livestock production systems, the extent of mammary gland development directly impacts the economic viability of the industry, as evidenced by strong positive correlations with key productivity metrics: lactation performance in mature dairy cows [82,83] and sow nursing capacity (reflected in piglet weaning weight) [84,85]. Notably, declining lactation performance and a high incidence of mastitis causing significant economic losses are challenges for the livestock industry. Therefore, research aimed at promoting mammary epithelial cell proliferation and differentiation, maintaining lactation homeostasis, and reducing mastitis through molecular regulation offers a promising avenue for addressing current challenges in the industry [86,87]. Within the ncRNA regulatory network, miRNAs have been shown to play a pivotal regulatory role in various aspects of animal development and cell differentiation [57]. In contrast, piRNAs have been identified as being essential for the formation of germ cells [73]. Although the precise functional mechanisms of piRNAs in mammary gland development remain to be fully elucidated, ample evidence demonstrates that miRNAs and lncRNAs in the ncRNA family play significant roles in mammary gland development and milk synthesis [88,89]. This underscores the multifaceted regulatory functions of the ncRNA family in mammary gland development.

As a prominent member of the short ncRNA family, miRNAs play a pivotal role in embryonic development [90], and their dysfunction has been associated with a range of diseases [91]. MiRNAs regulate gene expression through the sequence-specific recognition of the 3′ untranslated region (3′UTR) or other regulatory elements of target mRNAs. This regulatory mechanism exhibits unique network characteristics: a single miRNA can modulate the stability or translational efficiency of multiple target mRNAs, while a single mRNA may be concurrently regulated by several miRNAs, thereby orchestrating protein synthesis and signaling pathways [92]. Recent studies indicate that miRNAs serve as key regulators in the dynamic process of mammary gland development. The expression of miR-30b was found to be significantly increased in mice at the pubertal and maturity virgin stages. The overexpression of miR-30b resulted in reduced alveolar filling, smaller lumens, fewer lipid droplets, and structurally altered lipid droplets [93]. In addition, it was found that the process of mammary gland degradation was delayed. The expression of miR-31 increased progressively from the pubertal to the mature stages in mice. The inhibition of miR-31 expression resulted in a loss of mammary stem cells (MaSCS), fewer lipid droplets, and less alveolar tissue [94]. In goats, the expression of miR-103 was significantly higher at mid-lactation than at dry period. The overexpression of miR-103 promoted the accumulation of milk fat droplets and triglycerides in Goat Mammary Epithelial Cells (GMECs) [95]. Consequently, the expression patterns of miRNAs undergo characteristic changes throughout mammary gland development—including the formation of the embryonic mammary gland primordium, pubertal ductal morphogenesis, gestational alveolar proliferation, and functional maturation during lactation (Table 2). Collectively, these dynamic changes play a crucial regulatory role in every stage of mammary gland development. The livestock farming industry confronts a dual challenge of global population growth and an increasing demand for food. This dual challenge entails enhancing production efficiency and adopting green, circular development practices.

Although not as extensively studied as miRNAs, lncRNAs—another significant class of non-coding RNAs—have increasingly garnered research attention in recent years due to their essential roles in regulating mammary gland development. It has been demonstrated that the lncRNA SOX2OT harbors the transcription factor SOX2—one of the Yamanaka factors—which plays a pivotal role in embryonic development and is essential for maintaining the pluripotency of various stem cells. SOX2 has also been shown to be a key determinant [124]. Studies have demonstrated that steroid receptor RNA activator (SRA) significantly promotes the proliferation and differentiation of mammary epithelial cells through the synergistic activation of estrogen (ER) and progesterone (PR) receptors, while also triggering apoptosis. Furthermore, SRA enhances the progression of lobule–alveolar structures during pregnancy relative to control mice [89]. Zfas1 is expressed in developing a mammary gland after puberty, particularly in the epithelial cells of the ducts and alveoli during pregnancy. The knockdown of Zfas1 in HC11 cells has been shown to increase cellular proliferation, induce β-casein expression, and promote epithelial dome formation, suggesting that high Zfas1 expression in late gestational mammary glands may regulate proliferation and inhibit the terminal differentiation of alveolar cells [125].The H19 locus is regulated by estradiol and corticosterone, with high expression levels observed in alveolar cells during pregnancy and degeneration. H19 is developmentally regulated, exhibiting elevated transcript levels during both puberty and pregnancy [126]. Pregnancy-induced noncoding RNA (PINC) is a developmentally regulated lncRNA that is highly expressed in alveolar cells during pregnancy and in degenerating terminal ductal lobule-like structures during transplacental labor. PINC may inhibit the terminal differentiation of alveolar cells during pregnancy, thereby preventing the premature secretion of large quantities of milk [127,128].

Overall, ncRNAs play a pivotal role in the regulation of mammary gland development, with both miRNAs and lncRNAs having been demonstrated to exert a direct influence on this process. However, there remains a significant research gap regarding the specific mechanism of piRNAs in the development of the mammary gland. piRNAs have been demonstrated to perform distinctive functions in the context of reproductive development, and they also act as a member of ncRNAs. Consequently, it can be hypothesized that piRNAs may also be a pivotal factor influencing mammary gland development. Nevertheless, current research on piRNAs has been predominantly focused on model animals, and research on piRNAs in livestock is still limited.

## 5. Prospects for piRNAs in Livestock Animals

As members of ncRNAs, piRNAs play critical roles in numerous biological processes, including maintaining genomic stability, silencing transposons, and facilitating germ cell development. Recent research has revealed the potential significance of piRNAs in livestock species, with ongoing studies focusing on pigs, cattle, and sheep. Numerous investigations have characterized the expression profiles of piRNAs in various gonadal tissues across different developmental stages in livestock, offering novel insights into their roles in germ cell, embryonic, and overall gonadal development. In pigs, a “ping-pong” amplification loop of piRNAs has been proposed, suggesting that piRNAs repress transposons expression and regulate the post-transcriptional expression of multiple protein-coding genes critical for normal spermatogenesis, thereby enhancing our understanding of porcine spermatozoa development [129,130]. In cattle, hybrid male sterility (HMS) has been linked to promoter hypermethylation-induced silencing of PIWI/piRNA pathway genes. DNA methylation influences this pathway by affecting gene expression and the production of robust piRNAs during spermatogenesis, underscoring its central role in bovine HMS [131]. Studies have shown that piRNA expression in bovine frozen semen differs significantly between high-motility (HM) and low-motility (LM) sperm, suggesting that piRNAs may be involved in sperm development and overall fertility [132]. Additionally, the expression profiles of piRNAs in sheep ovaries during the luteal (LP) and follicular (FP) phases have been examined to provide a reference for understanding the role of ovarian piRNAs throughout the estrous cycle [133]. The results of the present study suggest that piRNAs play an integral role in domestic animals (Table 3). Moreover, analysis of milk exosomal ncRNAs has revealed the presence of 88 piRNAs of unknown function within milk, indicating a potential association between piRNAs and immune function [134]. Additionally, the antiviral defense function of piRNAs has been demonstrated in mosquito cells, and knocking down piRNA pathway proteins leads to the enhanced replication of the Semliki Forest virus, thereby underscoring the antiviral properties of the piRNA pathway. Consequently, the potential of piRNAs to enhance reproductive efficiency, optimize production performance, and augment disease resistance has been recognized. Compared with research on model organisms or humans, studies on piRNAs in animal husbandry are still in their infancy and warrant further investigation.

## 6. The Role of piRNAs in Breast Cancer

Although the precise mechanisms of piRNA action in mammary gland development remain ambiguous, recent analyses of piRNAs in human and mice have revealed defined endogenous piRNA expression patterns and mechanistic functions in breast cancer, with implications for regulatory control in mammary tissues [153]. Using small RNA sequencing facilitates the identification of differentially expressed piRNAs in both tumor and non-tumor tissues of breast cancer. For instance, Huang et al. reported that piR-4987, piR-20365, piR-20485, and piR-20582 are up-regulated in tumors, with elevated piR-4987 expression correlating with positive lymph node status [154]. In addition, Hashim et al. found that in breast cancer cells, piR-34377, piR-35407, and piR-36743 are up-regulated, whereas piR-36026, piR-36249, piR-36318, and piR-36712 are down-regulated—suggesting potential regulatory roles in the cell cycle, apoptosis, cell–cell interactions, and DNA replication and repair [155]. Other studies have identified differentially expressed piRNAs in various breast cancer types and following different treatments [156,157].

Further studies have revealed that piRNAs act through multiple pathways and clarified their molecular mechanisms. piR-651 is highly expressed in breast tumor tissues and cells, and binds to PIWIL2 to form a complex that promotes the DNMT1-mediated methylation of the phosphatase and tensin homolog (PTEN) promoter, which promotes cell proliferation and invasion and elevates MDM2, CKD4, and CyclinD1 protein levels, as well as inhibits apoptosis; the disruption of piR-651 results in the opposite effect [158]. Similarly, piR-823 has been shown to be upregulated in breast cancer cells and enhance the expression of stem cell regulators (OCT4, SOX2, KLF4, NANOG, h-TERT) and methyltransferases (DNMT1, DNMT3A, DNMT3B), thereby promoting the hypermethylation of the adenomatous polyposis coli (APC) promoter, activating Wnt signaling, and driving tumor growth—whereas piR-823 knockdown, which also increases ERα and decreases h-TERT expression via the inhibition of the PI3K/Akt/mTOR pathway, suppresses cancer cell proliferation [159,160]. In addition, piR-2158 is downregulated in human and rodent breast cancer tumors and inhibits interleukin-11 (IL-11) expression by competing with FOS-related antigen 1 (FOSL1), inactivates the JAK/STAT pathway, and suppresses cell proliferation, migration, epithelial–mesenchymal transition (EMT), stemness, and angiogenesis [18]. Collectively, these findings underscore the regulatory role of piRNAs in breast cancer (Table 4).

Given the above outlined mechanisms, piRNAs have shown a great deal of promise for therapeutic use. The use of piR-2158 nanoparticles as a therapeutic target for the treatment of breast cancer has been validated by their demonstrated significant inhibitory effects on the growth of mouse breast cancer tumors [18]. Additionally, piRNAs can be utilized as a potential molecular staging and prognosis tool and has been shown to be closely associated with breast cancer metastasis, staging, and response to treatment. Consequently, piRNAs may serve as both prognostic markers and therapeutic targets for breast cancer. However, more study is still required to accurately diagnose piRNAs and develop targeted therapies because our present understanding of the precise molecular pathways behind their significance in breast cancer is still restricted.

Beyond oncogenesis, numerous studies have highlighted critical roles for piRNAs in maintaining genome stability, regulating stem cell function, and modulating molecular signaling pathways. Many studies have demonstrated the pivotal role of the piRNA pathway in the maintenance, division, and differentiation of GSCs [172,173]. These findings offer important clues to their physiological roles in normal mammary gland development. Mammary gland development is a complex, multi-stage process encompassing the embryonic formation of the mammary anlage, pubertal ductal branching, and gestational differentiation of glandular structures [174]. The central roles of stem cells, hormonal stimulation, and signaling pathways underpin both normal development and breast cancer pathogenesis. Indeed, mammary stem cell MaSCs maintain tissue homeostasis and regeneration through self-renewal, whereas breast cancer stem cells (BCSCs) drive tumor growth and recurrence [174,175,176]. Moreover, both mammary development and breast cancer have been associated with the activation of key pathways—including Wnt, Notch, and Hedgehog—and with the pivotal influence of the estrogen receptor ERα in mammary morphogenesis and tumorigenesis [177,178,179]. These correspondences suggest that the physiological development of breast tissue shares common molecular characteristics with the pathological progression of breast cancer; accordingly, it is plausible that piRNAs contribute to the regulation of cell proliferation, differentiation, and homeostasis in normal mammary gland development.

## 7. The Role of piRNAs in Inflammation

The inflammatory response is characterized by the activation of immune cells, such as dendritic cells, neutrophils, and mast cells [180], and the release of pro-inflammatory factors, including tumor necrosis factor-alpha (TNF-α), IL-1, and IL-6 [181]. The role of non-coding RNAs (ncRNAs) in the regulation of inflammation has become a subject of considerable interest in the research community [182]. Among them, piRNA, as a significant member of the ncRNA family, is progressively being revealed to possess an inflammatory regulatory function in addition to its role in breast cancer. It has been shown that piRNAs are involved in the regulation of inflammatory responses through a multilevel molecular mechanism. First, piRNAs can play a regulatory role by directly acting on key inflammatory signaling pathways. For example, pir-has-216911 binds to Toll-like Receptors 4(TLR4) mRNA and suppresses the TLR4/NF-κB/NLRP3 inflammatory signaling pathway, thereby inhibiting caspase-1–induced activation of GSDMD and reducing the pro-inflammatory effects of pyroptosis [183]. Similarly, piR-112710 directly binds to the 3′UTR of the thioredoxin-interacting protein (Txnip), suppressing its expression and inactivating the Txnip/NLRP3 pathway; this leads to reduced levels of pro-inflammatory factors (IL-18 and IL-1β) and the lower expression of proteins related to inflammasome activation (NLRP3, caspase-1, and GSDMD-N) [19,184]. Furthermore, hsa_piR_019949 is significantly down-regulated in response to IL-1β, and it may modulate the inflammatory response by repressing the expression of the lncRNA NEAT1—which in turn lowers NLRP3 levels and regulates the NOD-like receptor signaling pathway [185]. Secondly, it has been demonstrated that certain piRNAs regulate inflammation-related genes through epigenetic modification pathways. It was found that piRNA-6426 has been shown to inhibit inflammation by increasing methylation at the SOAT1 promoter via the recruitment of DNMT3B, which reduces the secretion of IL-1β and TNF-α and ameliorates the inflammatory microenvironment in heart failure [186]. This epigenetic regulatory mechanism provides a new direction for the treatment of inflammatory diseases. Thirdly, in disparate cell types, piRNAs manifested particular regulatory functions. In endothelial cells, rno-piR-017330 is up-regulated in response to TNF-α stimulation, suggesting that piRNAs contribute to the regulation of inflammation in these cells [187]. In contrast, in chondrocytes, IL-1β promotes the expression of piRNA mmu_piR_037459, suggesting that inflammatory factors may drive osteoarthritis (OA) pathology by regulating piRNA levels [188]. Finally, clinical studies have revealed correlations between piRNA expression profiles and disease states. Saha et al. identified 19 differentially expressed piRNAs in the plasma of patients with chronic pancreatitis (CP) compared to healthy individuals [189]. This discrepancy in expression indicates that piRNA may be of pathophysiological significance in inflammatory diseases. It can thus be concluded that piRNAs also have an important role in the inflammatory response (Table 5).

Although the mechanisms by which piRNAs regulate inflammatory responses are gradually being elucidated, their specific role in mastitis—a common inflammatory condition of the mammary gland—remains unreported. Mastitis exhibits pathological similarities to other systemic or organ-specific inflammatory diseases (e.g., arthritis, pneumonia), providing a useful theoretical foundation for investigating shared mechanisms. The following three lines of evidence were used to formulate a hypothesis regarding the potential involvement of piRNAs in the regulation of mastitis in dairy cows through key pathways such as TLR4/NF-κB: First, mastitis and other inflammatory diseases share a similar immune microenvironment, characterized by the infiltration of inflammatory cells such as monocytes, dendritic cells, and macrophages that release reactive oxygen species (ROS) and proteases, thereby exacerbating tissue damage [192]. Second, key pro-inflammatory cytokines—namely, TNF-α, IL-1β, and IL-6—are elevated in mastitis, driving the inflammatory response and contributing to tissue destruction [71,193]. Third, mastitis shares essential inflammatory pathways such as NF-κB, the NLRP3 inflammasome, and MAPK with other inflammatory conditions [194,195,196]. Therefore, although the role of piRNAs in mastitis remains ambiguous, their established functions in regulating cytokine secretion, NLRP3 inflammasome expression, and NF-κB signaling in other inflammatory disorders suggest that piRNAs may similarly influence mastitis progression.

**Figure 3:**
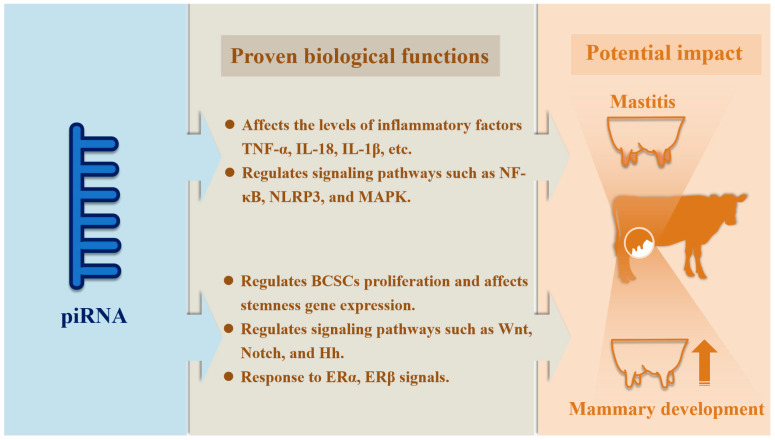
Possibility of piRNA in mammary development and mastitis.

In light of the functions of piRNAs in breast cancer progression and inflammatory responses, a comprehensive analysis was conducted regarding the potential roles of piRNAs in mammary gland development and mastitis (Figure 3). This investigation offers new insights into the advancement of animal husbandry.

## 8. Conclusions

‘Livestock are economically vital’ is punchier, providing a stable, high-quality supply of meat, eggs, and milk, while its scientific breeding mode is directly related to environmental safety, disease prevention, and biosecurity [197,198]. Despite their importance, research into the roles of piRNAs in livestock remains in its infancy, and systematic investigations are scarce. In this paper, we demonstrate that piRNAs are involved in numerous biological processes—including gamete formation, embryonic development, and disease regulation. Moreover, we propose that these small ncRNAs may influence livestock growth performance, disease resistance, and environmental adaptation through epigenetic mechanisms. At present, however, we are still unable to answer the questions “Can piRNAs regulate mammary gland development and can piRNAs be used as markers for mastitis in dairy cows?” Consequently, a comprehensive analysis of the molecular mechanisms underlying piRNA function in livestock is imperative to optimize breeding strategies, mitigate disease risks, and cultivate high-quality breeds—ultimately contributing to the industrial upgrading and high-quality development of animal husbandry. Notably, this study innovatively suggests that piRNAs might regulate the mammary developmental cycle and the pathological process of mastitis, offering new perspectives on enhancing lactation performance and developing innovative disease prevention and control strategies, which advance the development of dairy and animal husbandry in response to the dual challenges of a growing global population and increasing food demand.

## Figures and Tables

**Table 1:** Proteins involved in the generation of piRNAs in different species.

Species	5′-Terminal Enzyme	3′-Terminal Enzyme	Key PIWI Protein	Reference
Nematodes	Uncertainty	Uncertainty	Plasticity-related gene 1 (PRG-1), plasticity-related gene 2 (PRG-2)	[29]
Zebrafish	Phospholipase D family member 6 (PLD6)	Uncertainty	ZIWI, ZILI	[30,31]
Drosophila	Zuc	Trimmer	PIWI, Argonaute 3 (Ago3), Aubergin(Aub)	[32,33]
Mice	PLD6	Poly(A)-specific ribonuclease-like domain containing 1 (PNLDC1)	MIWI, MIWI2, MILI	[34,35,36]

**Table 2:** The role of miRNAs in mammary gland development.

Particular Year	miRNA	Research Target	Model Type	Outcomes	References
		Mammary gland development			
2007	let-7	Comma-Dβ	In vitro	Inhibited self-renewal capacity of progenitor cells and promoted differentiation.	[96]
2014	miR-21	HC11, mice	In vivo and in vitro	Regulated mammary gland development and lactation.	[97]
2012	miR-30b	Mice	In vivo	Inhibited normal mammary gland development and lipid droplet accumulation.	[93]
2020	miR-31	HC11, mice	In vivo and in vitro	Promoted MaSCS self-renewal, alveogenesis, and lipid droplet accumulation.	[94]
2020	miR-34a	Comma-Dβ, SUM159pt, mice	In vivo and in vitro	Inhibited MaSCs self-renewal, terminal end bud (TEB) development.	[98]
2009	miR-101a	HC11, mice	In vivo and in vitro	Inhibited HC11 proliferation and β-casein expression, affected mammary gland development and degeneration.	[99]
2010	miR-132, miR-212	Mice	In vivo	Promoted ducts growth and modulated epithelial–stromal interactions.	[100]
2015	miR-137	MDA-MB-231, 293T, mice	In vivo and in vitro	Promoted thickening of the mammary substrate.	[101]
2006	miR-138	Mouse mammary epithelial cells, mice	In vivo and in vitro	Regulated mammary epithelial cell proliferation and mammary gland development, promoted β-casein expression.	[102]
2017	miR-139	BMEC, Holstein cows	In vivo and in vitro	Inhibited β-casein synthesis and BMEC proliferation.	[103]
2022	miR-142-5p, miR-148C, miR-152, miR-218,	Goats	In vivo	Regulated mammary gland regenerative degeneration.	[104]
2014	miR-193b	MEC, mice	In vivo and in vitro	Inhibited mammary stem/progenitor cell activity and alveolar differentiation.	[105]
2009	miR-200c	293T, Tera-2, mice	In vivo and in vitro	Inhibited mammary duct formation.	[106]
2018	miR-205	MEC, mice	In vivo and in vitro	Impacted mammary regenerative capacity and mammary homeostasis.	[107]
2019	miR-489	Mouse mammary epithelial cells, mice	In vivo and in vitro	Inhibited duct growth and TEB formation.	[108]
		Milk component synthesis			
2018	miR-15b	MCF-10A, mice, goats	In vivo and in vitro	Inhibited lipid synthesis and metabolism.	[109]
2017	miR-17-5p miR-148a	GMEC, goats	In vivo and in vitro	Promoted triacylglycerol (TAG) synthesis and milk fat droplet accumulation.	[110]
2015	miR-24	GMEC, goats	In vivo and in vitro	Increased unsaturated fatty acid concentrations, TAG levels, and milk fat droplet accumulation.	[111]
2018	miR-25	GMEC, goats	In vivo and in vitro	Inhibited TAG synthesis and lipid droplet accumulation.	[112]
2013	miR-27a	GMEC, goats	In vivo and in vitro	Inhibited TAG synthesis and reduced the ratio of unsaturated/saturated fatty acids.	[113]
2015	miR-29s	DCMEC, 293T, Chinese Holstein cows	In vivo and in vitro	Inhibited triglyceride, protein, and lactose secretion.	[114]
2013	miR-103	GMEC, goats	In vivo and in vitro	Promoted lipid droplet accumulation and TAG accumulation.	[95]
2011/2017	miR-126-3p	MCF-10A, mice	In vivo and in vitro	Inhibited β-casein secretion and lipid synthesis.	[115,116]
2019	miR-142-3p	MMGEC, mice	In vivo and in vitro	Inhibited secretion of β-casein and TAG.	[117]
2017	miR-145	GMEC, goats	In vivo and in vitro	Promoted lipid droplet enlargement and TAG accumulation, increased the relative content of unsaturated fatty acids.	[118]
2016	miR-150-5p	Mice	In vivo	Inhibited the de novo synthesis of lipids and fatty acids.	[119]
2016	miR-181b	GMEC, goats	In vivo and in vitro	Increased TAG levels and cream droplet accumulation.	[120]
2020	miR-204	HC11, mice	In vivo and in vitro	Promoted β-casein and milk fat synthesis.	[88]
2019	miR-206	HC11, mice	In vivo and in vitro	Promoted lipid accumulation.	[121]
2018	miR-221	MEC, MCF-10A, mice	In vivo and in vitro	Promoted lipid synthesis.	[122]
2015	miR-486	BMEC, Holstein cows	In vivo and in vitro	Promoted beta-casein, lactose, and lipid secretion.	[123]

**Table 3:** The role of piRNAs in domestic animals.

Particular Year	Detection Methods	Species	Outcomes	References
2021	Small RNA-seq	Porcine	Characterization of the composition of piRNAs in spermatozoa suggests that piRNAs may be potential negative regulatory markers of sperm quality.	[135]
2012	Small RNA-seq, qRT-PCR	Porcine	It was demonstrated that piRNAs were predominantly enriched in the mature gonads and were expressed more in the testis than in the ovary.	[136]
2023	Small RNA-seq	Porcine	Expression of piRNAs is regulated by Senecavirus A (SVA) and promotes apoptosis.	[137]
2015	Small RNA-seq	Porcine	Characterization of the composition of piRNAs in testis suggests that mammalian piRNAs exist in the ping-pong cycle and have a role in the post-transcriptional regulation of protein-coding genes.	[130]
2022	Small RNA-seq	Xiang pigs	Identification of the composition of piRNAs in testicular tissues at different stages demonstrates that piRNAs regulate spermatogenesis.	[129]
2017	Small RNA-seq, qRT-PCR	Porcine	Characterization of the expression profiles of testicular piRNAs at different stages of sexual maturation demonstrated that piRNAs regulate testicular development and spermatogenesis.	[138]
2012	Small RNA-seq, qRT-PCR	Porcine	Evidence for a potential role of piRNAs in female germ cell development.	[139]
2020	Small RNA-seq, qRT-PCR	Porcine	Characterization of the expression profile of sperm plasma extracellular vesicles (SP-EVs) piRNAs suggests that piRNAs play a role in the physiological function of spermatozoa.	[140]
2017	Small RNA-seq, qRT-PCR	Bovids	The piRNAs in the testis were identified as longer than the piIRNAs in oocytes and embryos.	[141]
2020	Small RNA-seq	Yattle, cattle, yaks,	Promoter hypermethylation of PIWI/piRNA pathway genes leading to gene silencing and reduction in testis-thick piRNAs is a driver of bovine HMS.	[131]
2015	Small RNA-seq	Calves	Expression of piRNAs in bovine blood and plasma was revealed, suggesting that they may originate from tissues other than blood cells and thus enter the circulation.	[142]
2018	Small RNA-seq	Cattle, yaks, dzo	Comparison of the expression characteristics of three ruminant piRNAs provides theoretical references for exploring their regulatory mechanisms in spermatogenesis and dzo reproductive therapy.	[143]
2020	Small RNA-seq, qRT-PCR	Bulls	Expression of piRNAs in spermatozoa was detected, suggesting that they may play a role in embryonic development and may serve as biomarkers of semen fertility.	[144]
2017	Small RNA-seq	Bulls	Characterization of the composition of piRNAs in frozen spermatozoa suggests a role in sperm development and fertility.	[132]
2015	Small RNA-seq	Bovine	Detection of the composition of mature testicular and ovarian piRNAs revealed that ovarian piRNAs were very similar to spermatogenesis thick-walled stage piRNAs.	[145]
2018	Small RNA-seq	Bovids	Detection of milk exosomal piRNAs expression suggests that they may be related to immune and developmental functions.	[134]
2021	Small RNA-seq	Cattle	The presence of piRNAs in ejaculated sperm was confirmed, suggesting that they may regulate sperm maturation, fertilization process, and embryonic genome activation.	[146]
2021	Small RNA-seq	Bovids	Expression of piRNAs was detected separately in both milks, suggesting a possible regulatory role in calf immunity and development.	[147]
2023	Small RNA-seq	Murrah buffalo	Characterization of the composition of piRNAs at different stages of lactation implies that piRNAs can serve as potential targets for the regulation of lactation.	[148]
2019	Small RNA-seq	Mongolian horse	Characterization of piRNAs composition in sexually mature and immature testes suggests that piRNAs may regulate testicular development and spermatogenesis.	[149]
2022	Small RNA-seq	Sheep	Expression profiles of piRNAs in LP and FP ovaries were characterized to facilitate understanding of the role of piRNAs in the estrous cycle.	[133]
2022	Small RNA-seq	Sheep	Characterization of the composition of testicular piRNAs demonstrates that piRNAs may mediate blood–testis barrier stability and spermatogonial stem cell differentiation.	[150]
2021	Small RNA-seq	Sunite (SN), Small-tailed Han (STH)	Identification of differential expression of testicular piRNAs in different breeds suggests that piRNAs may be associated with male fecundity.	[151]
2023	Small RNA-seq, qRT-PCR	Tibetan sheep	Characterization of piRNAs expression profiles in different stages of testis suggests that piRNAs regulate male fertility and spermatogenesis.	[152]

**Table 4:** The role of piRNAs in breast cancer.

Particular Year	piRNA	Expression	Model Type	Species	Finding	References
2021	piR-651	Upregulation	In vivo and in vitro	Human	Bound to PIWIL2, promoted cell proliferation and migration through DNMT1-mediated methylation of the PTEN promoter.	[158]
2021	piR-823	Upregulation	In vivo and in vitro	Human and mice	Increased the expression of DNMT1, DNMT3A, and DNMT3B genes to promote DNA methylation of APC genes to activate the Wnt signaling pathway.	[159]
2022	piR-823	Upregulation	In vivo and in vitro	Human and mice	Inhibited piR-823 expression inhibited cell proliferation, PI3K/Akt/mTOR gene expression, and increased gene and protein expression of ERα.	[160]
2013	piR-932	Upregulation	In vivo and in vitro	Human and mice	Bound to PIWIL2, promoted methylation of promoter CpG islands to repress Latexin expression.	[13]
2023	piR-2158	Downregulation	In vivo and in vitro	Human and mice	Competed with FOSL1 to inhibit IL-11 expression and secretion, inactivating JAK/STAT signaling and thereby inhibiting breast cancer.	[18]
2022	piR-17560	Upregulation	In vivo and in vitro	Human	Targeted FTO-mediated m6A demethylation enhances ZEB1 expression, thereby promoting chemotherapy resistance and EMT.	[161]
2013	piR-4987, piR-20365, piR-20485, piR-20582	Upregulation	In vivo	Human	Influenced cancer development and lymph node metastasis.	[154]
2017	piR-1282, piR-21131, piR-23672, piR-26526, piR-26527, piR-26528, piR-30293, piR-32745	Upregulation	In vivo	Human	Can be used as a biomarker for breast cancer and provided a therapeutic target.	[156]
	piR-23662	Downregulation				
2014	piR-31106	Upregulation	In vivo and in vitro	Human	Responded to cell growth, cell cycle progression, and hormonal signaling.	[155]
2021	piR-31106, piR-34998, piR-40067	Upregulation	In vivo	Human	Can be used as a prognostic and therapeutic marker for breast cancer.	[157]
2023	piR-31106	Upregulation	In vivo and in vitro	Human	Promoted cell proliferation and migration as well as oncogene expression and METTL3-mediated m6A methylation.	[162]
2025	piR-31115	Upregulation	In vivo and in vitro	Human	Bound to PIWIL4 and inhibits the degradation of HSP90AA1 protein, thereby promoting cell migration.	[163]
2020	piR-31143	Upregulation	In vivo and in vitro	Human	Can modulation of TNBC behavior through ERβ.	[164]
2014	piR-34377, piR-35407, piR-36743	Upregulation	In vivo and in vitro	Human	Responded to cell growth, cell cycle progression, and hormonal signals.	[155]
	piR-36026, piR-36249, piR-36318, piR-36712	Downregulation				
2019	piR-36712	Downregulation	In vivo and in vitro	Human and mice	Knockdown of piR-36712 inhibits p53 activity through SEPW1, upregulates Slug/p21, and decreases E-calmodulin levels, ultimately inhibiting cell proliferation, migration, and invasion.	[165]
2020	piR-016658	Upregulation	In vivo and in vitro	Human	Regulated by cell Cyclin D1, affects stem cell function.	[166]
	piR-016975	Downregulation				
2015	piR-021285	Upregulation	In vivo and in vitro	Human	Increases the methylation level of the ARHGAP11A gene, which promotes cell invasion and inhibits cell apoptosis.	[167]
2015	piR-sno75	Upregulation	In vivo and in vitro	Human and mice	Binding to WDR5 recruits the MLL3/UTX complex to the TRAIL promoter region, thereby inducing H3K4 methylation and H3K27 demethylation.	[168]
2022	piR-MW557525	Upregulation	In vivo and in vitro	Human	Promotes the proliferation, migration, and invasion of Piwil2-iCSCs, promotes the expression of CD24, CD133, KLF4, and SOX2, and inhibits apoptosis.	[169]
2018	piR-FTH1	Downregulation	In vivo and in vitro	Human	Binds to HILI/HIWI2 and down-regulates FTH1, increasing sensitivity to chemotherapy.	[170]
2024	piR-YBX1	Downregulation	In vivo and in vitro	Human and mice	Inhibition of YBX1 expression leads to inhibition of MEK and ERK1/2 MAPK signaling pathways, ultimately inhibiting cell proliferation and migration.	[171]

**Table 5:** The role of piRNAs in inflammation.

Particular Year	piRNA	Expression	Research Target	Finding	References
2024	hsa-piR-3411, hsa-piR-24541, hsa-piR-27080, hsa-piR-28104, hsa-piR-32157 and 10 others	Upregulation	Human peripheral venous blood	Identification of piRNAs in the plasma of CP patients demonstrated that piRNAs are associated with inflammation.	[189]
	hsa-piR-32835, hsa-piR-32836, hsa-piR-32986, hsa-piR-33168	Downregulation			
2022	piRNA-6426	Downregulation	Rat cardiomyocytes, rats	Inhibits secretion of inflammatory factors IL-1β and TNF-α, cardiomyocyte apoptosis, oxidative stress, and improves the inflammatory microenvironment in heart failure.	[186]
2023	piR-has-27620, piR-has-27124	Upregulation	Blood samples	Identification of peripheral leukocyte piRNA expression and their enrichment in Rap1, PI3K-Akt, and MAPK pathways as RA biomarkers.	[190]
2021	rno-piR-017330	Upregulation	Endothelial cells, rats	Identification of piRNAs expression in endothelial cells under inflammatory conditions suggests that piRNAs may regulate inflammatory processes.	[187]
2024	hsa_piR_019949	Downregulation	C28/I2, SW1353	Inhibition of NEAT1 and NLRP3 expression regulates the NOD-like receptor signaling pathway and modulates OA progression.	[185]
2024	mmu_piR_037459	Upregulation	Mice cardiomyocytes, mice	Inhibition of collagenase II expression, promotion of chondrocyte apoptosis and inhibition of proliferation, inhibition of USP7 expression, and regulation of OA progression.	[188]
2024	piR-112710	Downregulation	Mice cardiomyocytes, mice	Inhibits the Txnip/NLRP3 signaling pathway, reduces the levels of IL-18, IL-1β, and NLRP3, inhibits cardiomyocyte injury, and regulates inflammation progression.	[19]
2025	pir-has-216911	Upregulation	HL7702, Huh7, HepG2, Hep3B, nude mice	Inhibition of the TLR4/NFκB/NLRP3 inflammatory signaling pathway suppressed the inflammatory response.	[183]
2020	piRNAs	Differential expression	Sudani duck	The composition of piRNAs in brain and lung was characterized, suggesting that they may be associated with lung inflammation.	[191]

## Data Availability

The original contributions presented in this study are included in the article. Further inquiries can be directed to the corresponding authors.
